# Nanomedicines in diagnosis and treatment of prostate cancers: an updated review

**DOI:** 10.3389/fbioe.2024.1444201

**Published:** 2024-08-21

**Authors:** Jiajia Wang, Xuan Zhang, Jiazhen Xing, Lijian Gao, Hua Lu

**Affiliations:** ^1^ Department of Oncology, Qilu Hospital of Shandong University Dezhou Hospital, Dezhou, China; ^2^ Department of Urology, Qilu Hospital of Shandong University Dezhou Hospital, Dezhou, China

**Keywords:** prostate cancer, nanomedicine, exosome, diagnosis, therapeutic application

## Abstract

Prostate cancer (PC) is the third most common male cancer in the world, which occurs due to various mutations leading to the loss of chromatin structure. There are multiple treatments for this type of cancer, of which chemotherapy is one of the most important. Sometimes, a combination of different treatments, such as chemotherapy, radiotherapy, and surgery, are used to prevent tumor recurrence. Among other treatments, androgen deprivation therapy (ADT) can be mentioned, which has had promising results. One of the drawbacks of chemotherapy and ADT treatments is that they are not targeted to the tumor tissue. For this reason, their use can cause extensive side effects. Treatments based on nanomaterials, known as nanomedicine, have attracted much attention today. Nanoparticles (NPs) are one of the main branches of nanomedicine, and they can be made of different materials such as polymer, metal, and carbon, each of which has distinct characteristics. In addition to NPs, nanovesicles (NVs) also have therapeutic applications in PC. In treating PC, synthetic NVs (liposomes, micelles, and nanobubbles) or produced from cells (exosomes) can be used. In addition to the role that NPs and NVs have in treating PC, due to being targeted, they can be used to diagnose PC and check the treatment process. Knowing the characteristics of nanomedicine-based treatments can help design new treatments and improve researchers’ understanding of tumor biology and its rapid diagnosis. In this study, we will discuss conventional and nanomedicine-based treatments. The results of these studies show that the use of NPs and NVs in combination with conventional treatments has higher efficacy in tumor treatment than the individual use of each of them.

## 1 Introduction

Prostate cancer (PC) is one of the most common cancers in men (second in the United States), which mostly have an inactive period. The mortality rate in patients with this disease is high (Ye et al., 2022), and most localized prostate cancers have an indolent course and lead to deaths within 15 years due to other causes, even with current follow-ups and treatments (Popiolek et al., 2013). One of the main reasons that lead to the formation of this cancer is genomic alterations that occur in prostate cells. It seems that the genome market is caused by disturbances in the structure of chromatin or impaired transcription and can ultimately lead to the creation of prostate tumors (Berger et al., 2011). ERG, PTEN, and MAGI2 can be mentioned among the genes whose expression disorder can lead to prostate cancer (Carver et al., 2009). Also, examining the state of TMPRSS2-ERG fusion can be used as a prognostic factor of tumor type (Markert et al., 2011).

According to the model presented by Logothetis et al. (2013) PC development is divided into three categories. In the first category, which is related to the endocrine glands, the signaling of androgens is essential. It happens after dihydrotestosterone (DHT) decreases, known as the endocrine stage. In the next stage and after escaping from dependence on DHT, which is the stage dependent on the microenvironment, paracrine signaling becomes more critical; however, androgen signaling is still essential. In the final stage, androgen dependence is lost, and tumor cells can survive and reproduce without needing this hormone (Logothetis et al., 2013). It seems that this type of cancer modeling can determine the different stages of PC and its effective treatments.

Different treatments are used according to the tumor stage; however, they are divided into four categories based on the path they affect (Swami et al., 2020). The first category, including enzalutamide, darolutamide, abiraterone, and apalutamide, are drugs that affect the androgen axis and lead to the management of advanced prostate cancer (Ryan et al., 2015; Fizazi et al., 2019; Hoyle et al., 2019). The second, including cabazitaxel and docetaxel, has therapeutic effects on this cancer by affecting the polymerization and depolymerization of microtubules and mitosis and cell proliferation (James et al., 2016; Sydes et al., 2018). Another type of medicine that includes chemotherapy and radioactive materials can be used in the treatment of many cancers. Other treatments affect the immune system and its mechanisms and increase its response to tumor cells.

In the meantime, some drugs that have a small size in the range of nanometers are getting more attention nowadays. Because of their small size, ability to pass through the blood-tumor barrier and stable surface function, these drugs are suitable choices for tumor treatment (Lakshmanan et al., 2021). Also, nanocarriers, including exosomes, liposomes, micelles, and dendrimers, lead to overcoming the disadvantages of conventional treatments and drugs and increase bioavailability and targeted therapy (Rana et al., 2023). Based on the results of various studies, nanoparticles can lead to the treatment of tumors through multiple mechanisms (Qi et al., 2020). One of these mechanisms is related to increasing the efficiency of immune checkpoint inhibitor-based treatments in combination with nanoparticles (Cheng et al., 2018; Zou et al., 2018). Also, using nanoparticles can affect immune cells’ function, including natural killer (NK) and T cells, and leads to an increase in the tumor-killing function of these cells (Zhou et al., 2019). Another cell that can be affected by nano-based treatment is dendritic cells (DCs) (Kroll et al., 2019). Studies have shown that some specific nanoparticles can increase the antigen-presenting potential of DCs and lead to increased activation. Therefore, besides the direct effect of nanoparticles, nanocarriers, and other nanotechnology-based drugs, these treatments can help treat tumors by affecting the immune system cells (Zheng et al., 2019).

Considering that nanomedicine has increased the efficiency of immunotherapy and the use of immune system mechanisms (Irvine and Dane, 2020; Sun et al., 2020), this type of treatment is recommended for prostate cancer treatment. Although the results in the field are promising, examining treatments based on nanomaterials can help increase researchers’ understanding and open new doors to treating PC.

In this review, we will first talk briefly about the usual treatments for PC. We will mention nanomedicine-based methods for diagnosing and treating this disease and try to fully explain the different mechanisms involved in the therapeutic potential of these types of treatments.

## 2 Different treatments for PC

### 2.1 Androgen deprivation therapy (ADT)

Luteinizing hormone-releasing hormone (LHRH) agonists are one of the leading agents based on ADT, which has been used for years to treat cancers (Liu et al., 2011). LHRH receptor is expressed on the surface of many cancer cells, including PC cells (PCC) (Halmos et al., 2000). In the second half of the 20th century, LHRH agonists were widely used to treat PC (Tolis et al., 1982). The results of these studies show that the acute administration of LHRH agonists by stimulating the release of gonadotropins, including LH and FSH, from the pituitary gland leads to an increase in the concentration of testosterone and the functions of Lydic cells (Rick et al., 2013). However, chronic administration of LHRH agonists through desensitization and reduction of LHRH receptors leads to the suppression of Lydic and pituitary cell function (Rick et al., 2013). This treatment avoids surgery, leading to castration in many cases, and has high benefits. However, this type of treatment has side effects, including “Flare” effects, sexual effects, bone effects, cardiovascular disease and diabetes, hematological effects, and cognitive and emotional effects, and this leads to limiting the use of this type of drug (Seidenfeld et al., 2000; Van Poppel and Klotz, 2012; Rick et al., 2013).

Enzalotamide is another drug of the ADT family that is used as the standard first line of PC (Hoffman-Censits and Kelly, 2013). This drug is oral, and compared to docetaxel, which is one of its family drugs, it shows better clinical results in receiving patients (Merseburger et al., 2015). This drug works by inhibiting the function of androgen receptors and inhibiting the signaling of these receptors (Claessens et al., 2014). Among the side effects of this medicine are diarrhea, fatigue, hot flashes, and, in some cases, seizures (Hoffman-Censits and Kelly, 2013). Darulotamide is a 2nd generation androgen receptor inhibitor, which, due to its inability to cross the blood-brain barrier, has a meager chance of convulsions in patients who receive it (Abbasi et al., 2021). In addition, darulotamide can also inhibit the activity of several androgen receptor mutants resistant to enzalutamide (Abbasi et al., 2021). Darulotamide has therapeutic efficacy in lower doses than enzalutamide. Also, this drug does not lead to an increase in testosterone, and it seems that it does not have the side effects caused by the increase in testosterone by other ADTs.

Considering the side effects observed in patients receiving ADT, it is expected that the metabolic changes caused by these drugs are the leading cause of these side effects. The results of a study examining the serum of patients receiving ADT confirm these changes (Chi et al., 2020). The results show that ADT treatment leads to a decrease in the synthesis of steroids, which reflects the reduction of steroid hormones and androgen sulfate measured. In addition, as mentioned earlier, due to the development of diabetes in patients receiving ADT, the blood glucose level increases significantly (Chi et al., 2020). Also, ketogenesis, 3-hydroxybutyric acid, acyl-carnitines (resulting from fatty acid metabolism), and 3-formyl indole (resulting from tryptophan metabolism by microbiota) are reduced in patients receiving ADT (Chi et al., 2020).

The use of ADT may eventually lead to the spread of treatment-resistant PCC and castration-resistant prostate cancer (CRPC) with a poor prognosis (Konoshenko et al., 2021). However, ADT-based drugs have side effects that limit their use and require the use of newer treatments. Table 1 summarizes combined therapeutic approaches by emphasizing ADT.

**TABLE 1:** Example and importance of different treatments and their combinations for prostate cancer as an active field in clinical trials.

Study name	Estimated enrollment	Therapeutic intervention	Phase	Date	Status	NTC number
An open label phase II study of biweekly docetaxel plus androgen-deprivation therapy in patients with previously-untreated, metastatic, prostatic adenocarcinoma	42	1. Androgen-deprivation therapy (ADT) 2. Docetaxel	Phase 2	2022	Complete	NCT03061643
Neoadjuvant androgen deprivation, darolutamide, and ipatasertib in men with localized, high risk prostate cance	6	1. Ipatasertib 2. Darolutamide 3. ADT	Phase 1 Phase 2	2022	Terminated	NCT04737109
Darolutamide in addition to ADT versus ADT in metastatic hormone-sensitive prostate cancer	662	1. Darolutamide 2. ADT	Phase 3	2021	Active not recruiting	NCT04736199
Salvage radiotherapy combined with androgen deprivation therapy (ADT) with or without rezvilutamide in the treatment of biochemical recurrence after radical prostatectomy for prostate cancer	102	1. Salvage radiation therapy (SRT) 2. ADT 3. Rezvilutamide	Phase 2	2024	Recruiting	NCT06305832
n efficacy and safety study of enzalutamide plus androgen deprivation therapy (ADT) versus placebo plus ADT in chinese patients with metastatic hormone sensitive Prostate cancer	180	1. ADT 2. Enzalutamide	Phase 3	2023	Active not recruiting	NCT04076059
A study of apalutamide plus androgen deprivation therapy (ADT) versus ADT in participants with mHSPC	1,052	1. ADT 2. Apalutamide	Phase 3	2024	Active not recruiting	NCT02489318
Radioablation with or without androgen deprIvation therapy in metachronous prostate cancer oligometaStAsis	150	1. ADT 2. Radiation: SBRT	Phase 2	2023	Recruiting	NCT03940235

### 2.2 Chemotherapy

Chemotherapy is one of the approved and tried treatments for PC, and it is effective in different stages of PC treatment (Quinn et al., 2017). The use of this type of drug began in 1980, and the first approved drug of this family is mitoxantrone, which was approved in 1999 for PC treatment (Eisenberger et al., 1985). Mitoxantrone is a doxorubicin analog and a synthetic anthracenedione, so it is an improved drug compared to doxorubicin (Fox, 2004). It is essential to mention that the cardiac toxicity potential of mitoxantrone has decreased compared to doxorubicin (Durr et al., 1983). This drug accumulates quickly in tissues such as the heart, thyroid, and liver, and its half-life varies from 9 h to 9 days, depending on the tissue (An and Morris, 2012). This medicine helps treat tumors through different mechanisms. Among these mechanisms, one can suppress the function of immune system cells, including macrophages and B and T cells (decreasing proliferation, antibody production, and increasing regulatory activity), preventing the expression of surface antigens and the production of pro-inflammatory cytokines (Fox, 2004). In addition to cancer, this drug has also been used to avoid the worsening of multiple sclerosis (MS) (Scott and Figgitt, 2004). Mitoxantrone seems to perform this action by preventing myelin degradation by macrophages (Neuhaus et al., 2006). However, nowadays, this drug is of little application for PC treatment due to the long-term study results that have shown mitoxantrone cannot significantly increase the patient’s overall survival (Berry et al., 2002).

The following chemotherapy drug approved by the US Department of Health and Human Services in 2004 for treating PC is a semisynthetic taxane called docetaxel (Petrylak et al., 2004). Due to its antimitotic property, this drug is used together with prednisone as the first line of chemotherapy for CRPC (McKeage, 2012). It has also been shown that docetaxel can exert its therapeutic action by inhibiting microtubule polymerization (inducing arrest in the G2M phase of the cell cycle) and reducing survival, causing cell death in tumor cells by inhibiting the expression of Bcl-2 and Bcl-xL (Pienta, 2001). The results of studies have shown that docetaxel has a higher affinity for tubulin than taxane and inhibits mitosis in tumor cells with a higher potential (Crown et al., 2004). In addition, compared to paclitaxel, it has a higher potential to induce Bcl-2 phosphorylation (Crown et al., 2004). Therefore, this drug suppresses tumor growth by combining mitosis and survival inhibition. However, this drug also has side effects that limit its use. These complications include febrile neutropenia, nail changes, fluid retention, and hypersensitivity (Baker et al., 2009).

Cabazitaxel, as a second-line agent, is a second-generation taxane after docetaxel and was first approved in 2010 by the US Department of Health and Human Services and the Food and Drug Administration (FDA) for the treatment of PC in people previously treated with docetaxel containing chemotherapy regimens (Galsky et al., 2010; Quinn et al., 2017). Cabazitaxel is usually used for docetaxel-resistant PC treatment (Abidi, 2013). Cabazitaxel seems to be an alternative to mitoxantrone for most patients. Like docetaxel, cabazitaxel inhibits mitosis and cell cycle by binding to tubulin. Still, unlike docetaxel, it binds to P-glycoprotein (P-gp), which is a drug efflux pump (ATP-dependent) expressed by cancer cells, which leads to less drug resistance (Paller and Antonarakis, 2011). Therefore, the responsible and primary mechanism of using cabazitaxel instead of docetaxel is its non-depletion by P-gp. The result of the study conducted in 2019 shows that the use of cabazitaxel in the treatment of metastatic CRPC has a higher therapeutic efficiency than ADR-based treatments such as abiraterone and enzalutamide and significantly increases the overall survival of patients (de Wit et al., 2019). Among the side effects of cabazitaxel are neutropenia and diarrhea, while neuropathy was rarely observed, unlike other chemotherapy drugs (Paller and Antonarakis, 2011).

### 2.3 Surgery

Although most treatments for early-diagnosed PC include active surveillance through therapies such as ART, chemotherapy, and radiotherapy, some patients benefit from locally invasive treatments such as surgery (Sriprasad et al., 2009). Radical prostatectomy has advantages such as reducing mortality and increasing the survival of patients without experiencing metastasis (Walsh and Jewett, 1980). Radical prostatectomy includes removal of the entire prostate, pelvic lymphadenectomy, and seminal vesicles (Bill-Axelson et al., 2005). However, surgery or prostatectomy has complications such as erection problems and adverse effects on urinary control (Sebesta and Anderson, 2017). Therefore, the stage of the disease, the patient’s preferences, and the errors caused by it determine the type of the disease. The main goal of radical prostatectomy is to control cancer. Also, studies show that a better imaging technique in the preoperative environment can facilitate surgical planning (Checcucci et al., 2019). It also seems that the use of newer tools can help surgeons to perform prostatectomy more accurately. However, maintaining urinary control and not causing erection problems is very important.

### 2.4 Immunotherapy

Treatments based on immunotherapy use mechanisms that increase the ability of the immune system to fight against tumor cells (Del Paggio, 2018). As mentioned before, tumor cells prevent the killing of tumor cells by forming a complex microenvironment by producing immunosuppressive cytokines, increasing the expression of molecules related to immune checkpoints, and producing other soluble factors (Whiteside, 2006). Therefore, the use of strategies that can strengthen the immune system and enhance their tumor-killing potential is included in the category of immunotherapy and can help in the regression and treatment of PC. To date, two immunotherapy-based treatments for PC have received FDA approval, including Sipuleucel-T (Provenge^®^) and Dostarlimab (Jemperli) (Wang et al., 2022).

One of the main treatment strategies based on immunotherapy is blocking immune checkpoints. Dostarlimab is a monoclonal IgG4 antibody that binds to the programmed death receptor-1 (PD-1) on the surface of PC cells (Ali et al., 2022; Haarberg, 2022). It prevents interaction with its ligand on the surface of immune cells, especially T cells. The binding of this raptor ligand leads to the suppression of T-cell responses. Therefore, blocking this interaction can increase the ability of T cells to kill tumor cells (Farzeen et al., 2024). This type of treatment can be suitable for metastatic PCs. Pembrolizumab (Antonarakis et al., 2020), pembrolizumab plus docetaxel, and prednisone (Evan et al., 2022), or in combination with cryotherapy (Ross et al., 2020), can be used from other antibodies that are against PD-1 and have been used for the treatment of PC. In addition to PD-1, other ICBs can also be used to treat CP. For example, in various clinical trials, CTLA-4-binding ipilimumab (Fizazi et al., 2020), CTLA-4-binding tremelimumab (McNeel et al., 2012), as well as the combination of GM-CSF with ipilimumab (Fong et al., 2009), or the combination of radiotherapy with ipilimumab have been used to treat PC (Fizazi et al., 2020).

Another treatment based on immunotherapy is the use of cytokines that stimulate the immune system (Mao et al., 2021). Cytokines can be used directly and as a single therapy. IL-2, IL-12, IFN-γ, and GM-CSF are among the most important cytokines used in treating PC (Belldegrun et al., 2001; Ko et al., 2004; Tazaki et al., 2011). However, this type of use can lead to many side effects, including uncontrollable activation of immune cells through a positive feedback system.

GVAX is another cytokine-based treatment that can help treat PC by strengthening the immune system. In this type of cell treatment, PCCs undergo genetic engineering and find the ability to produce cytokines that stimulate the immune system, such as GM-CSF (Simons and Sacks, 2006).

Adoptive Cell Therapy (ACT) is another immunotherapy-based treatment that can be used for PC immunotherapy (Rosenberg et al., 2008). Sipuleucel-T, as an FDA-approved treatment for PC, is a cell-based autologous vaccine that, after extracting the patient’s own cells from their blood using prostatic acid phosphatase by antigen-presenting cells, which is a specific antigen for PC, T cells isolated from specifically activate the patients and then inject the patients (Kantoff et al., 2010). It seems that this treatment increases the problem of priming T cells and increases their ability to kill tumors after they are injected into the patient. Chimeric antigen receptor-expressing (CAR) T cells can be mentioned among these cells (Wolf et al., 2021). This type of T cell has a surface receptor that binds to a specific antigen on the surface of PCCs, leading to T cell activation (Schepisi et al., 2019). Among the CAR T used for treating PC, we can mention the CAR T expressing the specific receptors for epithelial cell adhesion molecule (EpCAM) (Bębnowska et al., 2020), NKG2D (He et al., 2020), and PSMA (Wang et al., 2022).

Bispecific T cell engager (BiTE) is another immunotherapy available for PC. It consists of an antibody with two parts of Fab with a linker that specific for two types of antigens, one on the tumor cell surface and one on the T cell surface (Goebeler and Bargou, 2020). One treatment is related to the specific BiTE of PSMA and CD3, which leads to the activation of T cells near tumor cells and increases their ability to kill tumors (Hummel et al., 2021).

## 3 Nanomedicine for PC diagnosis and treatment

In general, nano-sized materials are used to treat tumors in this type of treatment. These treatments include many materials, including nanoparticles (polymeric, silica, gold, and magnetic), drug-carrying vesicles (liposomes, micelles, nanobubbles, and exosomes), dendritic polymers, and quantum dots. Among the things that can exist regarding the difference in the therapeutic potential of these items include the size, electric charge, and surface properties of the particles used, which affect their biological distribution in the patient’s body. Among the most essential categories of nanomedicines are nanocarriers, which include double-layer or single-layer membranes, including liposomes, micelles, and exosomes, which, by enclosing medicinal substances, prevent them from breaking down in the blood and preventing them from being harmed.

Nanomedicine based on nanoparticles (NPs) has advantages over other treatments, including their small size, biocompatibility, and drug delivery ability. Also, nanovesicles were biocompatible; the possibility of targeting their migration to the tumor site and high ability to carry different drugs was pointed out. However, various studies have shown that nanoparticles are reduced to 15% of the injected amount in the bloodstream approximately 1 h after injection (Wen et al., 2023). Nanovesicles (NVs) may be removed from the blood circulation through macrophages or the reticuloendothelial system, and the effectiveness of their treatment will decrease (Tang et al., 2019). For this reason, engineering mechanisms have been used in many NPs and NVs to increase their therapeutic efficiency. Among these methods, we can mention the polymer-based NPs, the use of inorganic materials-based systems, bioinspired methods, and engineered lipid vesicles for targeted migration (Boisseau and Loubaton, 2011). These methods increase stability, improve pharmacokinetics and tissue distribution, increase targeted migration, and enhance nanobiological interactions of nanomedicine-based treatments (Li and Kataoka, 2020).

**FIGURE 1:**
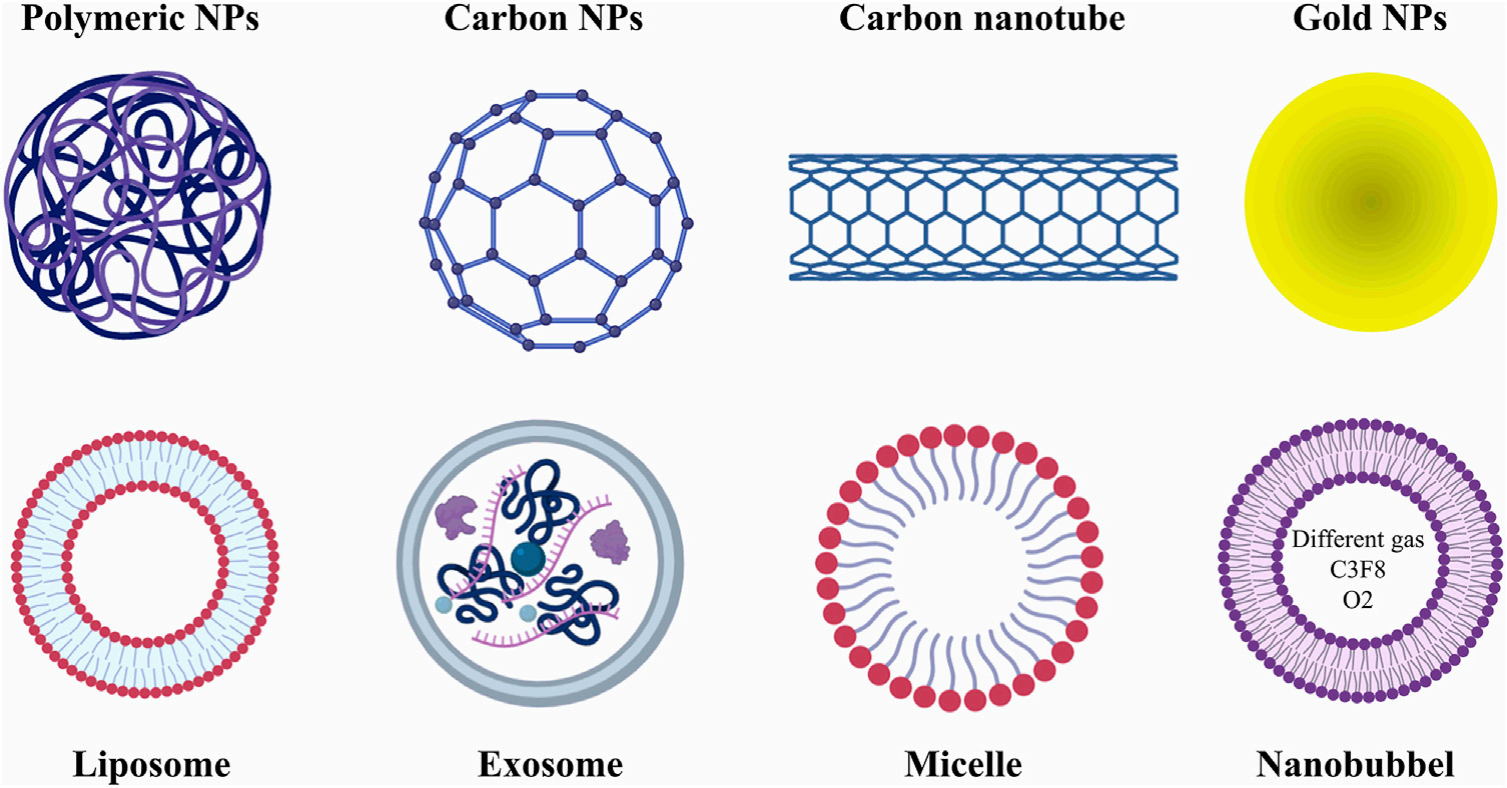
Different types of treatments based on nanomedicine. In general, nano treatments in this article are divided into two categories: nanoparticles (NPs) and nanovesicles (NVs). NPs play a very important role in the better diagnosis of prostate cancer; however, they can also be used as carriers of chemotherapy drugs. NVs are also widely used as drug carriers. However, it seems that nanobubbles play a greater role in improving tumor imaging contrast.

In addition, it seems that the use of nanocarriers can reduce the side effects of chemotherapy drugs, increase drug solubility, correct the biodistribution of the drug, have less renal elimination, and also due to the presence of abnormal vessels accumulate in cancerous tissues. It has also been shown that the specified ligand can be anchored with nanocarriers and bind to the overexpressed site of cancer cells for targeted drug delivery. NPs and NVs have a size of 1–1,000 nm, and based on their type, they are classified into different classes, including ceramic nanoparticles, carbon-based nanoparticles (fullerenes), polymer nanoparticles, and metal nanoparticles (Figure 1). Many of these nanoparticles have high potential in therapeutic applications and have been used in various studies (Table 2).

**TABLE 2:** Examples of NPs, their targeted molecules, and drug loading.

NP type	Therapeutic or diagnostic	Loaded drug	Ligand on NP	Target on cancer cell	Result	Ref
PNP (PLGA-PEG polymer)	Therapeutic	Docetaxel	PSMA aptamer	PSMA	1. ↑ Targeted delivery of docetaxel 2. ↑ Tumor cell apoptosis	Byrne et al. (2008)
	Therapeutic	Toremifene	Anti-PSMA antibody	PSMA	1. ↑ Tumor necrosis 2. ↑ Toremifene uptake by tumor cells 3. ↓ Growth of prostate tumor and proliferation	Hariri et al. (2015)
	Therapeutic	Docetaxel	ACUPA	PSMA	↑ Targeted delivery of docetaxel	Atkinson et al. (2018)
CNTs	Diagnostic	Thionine (electrochemical probe)	Anti-PSA antibody	PSA	Linear behavior of PSA concentrations was detected between 0.2 and 1 ng/mL and 1–40 ng/mL	Salimi et al. (2013)
	Diagnostic	NA	Goat-anti-rabbit IgG	Universal	Detect 8 pg/mL of IL-8 and 5 pg/mL of PSA in patient’s serum	Wan et al. (2011)
	Therapeutic	Carboplatin	NA (*In vitro*)	NA	↑ Accumulation of the chemotherapeutic drugs in cancer cells	Ringel et al. (2014)
Silver NP (AgNPs)	Therapeutic	Berberis thunbergii leaf extract	NA (*In vitro*)	NA	Dose-dependent toxicity on cancer cell	Guo et al. (2022)
AuNPs	Diagnostic	NA	Bombesin	gastrin-releasing peptide (GRP)	↑ Quality of molecular imaging via X-ray	Chanda et al. (2010)

### 3.1 Polymer-based system

Polymer nanoparticles (PNPs) have been used in PC treatment, and their results have been promising. PNPs have various characteristics, such as biocompatibility, appropriate biodistribution, and biodegradability, and can affect the pharmacokinetic properties of active substances (drugs). In addition, PNPs have features such as stability in complex environments such as tumor microenvironment (TME), control of size, flexibility in synthesis, and simultaneous delivery of several medicinal substances. TME includes all existing cells, soluble factors, and intercellular communication, which creates an environment for tumor expansion (De Visser and Joyce, 2023; Mayer et al., 2023). PNPs usually consist of two components. The first is the core of the NPs, which can be solid or liquid, and the next part is the polymer cortex. Drugs are usually either dissolved in the core or they can be connected to their polymer cortex through molecular bonds. Among the polymers used for the synthesis of PNPs, synthetic polymers (Poly lactic-co-glycolic acid (PLGA) and polylactide) and natural polymers such as gelatin, albumin, alginate, and chitosan can be mentioned, which are placed on the surface of nanoparticles. Medicines that these nanoparticles can be used in the treatment of prostate cancer, including chemotherapy drugs [doxorubicin (DTX) and quercetin (QU)] or LHRH ligands that are linked to PLGA/PEG with NPs.

The results of various studies have shown that the presence of LHRH on the surface of PNPs leads to an increase in their uptake by tumor cells. Therefore, it seems that the combined use of PNPs, which carry chemotherapy drugs DTX and QU and have LHRH on their surface, can target PCCs and suppress the growth of tumor cells (Shitole et al., 2020). In a study conducted in 2022 by Goswami et al. (2022) it has been shown that lycopene loaded in polymer nanoparticles can lead to the cumulative release of the drug as well as the reduction of proliferation in androgen-insensitive PC-3 prostate cancer cell lines and LNCaP cells become sensitive to androgens. Also, in the study conducted by Raspantini et al. (2021) they used polymeric nanoparticles based on polycaprolactone-DL-α-tocopherol-PEG-1000 copolymer encapsulated with docetaxel (Guo et al., 2013; Choudhury et al., 2017), which is a chemotherapy drug, for the treatment of PC. This study also showed that the PCL-TPGS polymer nanoparticle produced significantly leads to cell death and internalization in the PC-3 cell line. In addition, the results of the *in vitro* phase of this study show that the volume of induced tumors in mice has decreased compared to the control group (Raspantini et al., 2021).

Also, in some studies, PNPs have been used to increase the effectiveness of proven treatments. In the study conducted by Zean Li and colleagues, a drug called NSC23766, which has promising results *in vitro* (Levay et al., 2013) but showed low effectiveness *in vivo*, PNPs have been used to increase the efficiency of this drug (Li et al., 2022). This study used polymers based on L-phenylalanine poly (esteramide) (Phe-PEA) to cover NPs, ultimately producing NSC23766@8P6 nanoparticles. The results of the *in vitro* phase showed that the use of this nanoparticle is absorbed by PC3 cells at a high speed, and through the effect on the G2/M phase of mitosis, it leads to a decrease in the proliferation of cancer cells. Also, intravenous (IV) injection of NSC23766@8P6 nanoparticles *in vivo* to the mouse model of prostate cancer leads to a decrease in tumor growth, an increase in the apoptosis of cancer cells, and a reduction in tumor size (Li et al., 2022). In another study, nanoparticles coated with an amphiphilic ternary copolymer consisting of PEG, PLGA, and Wy5a aptamer encapsulated with docetaxel were used to treat PC (Fang et al., 2020). The result of this study shows the controlled release of DTX and the increase of their ability to kill tumors *in vitro*. Also, *in vivo* investigations for PC treatment show the absence of systemic drug toxicity and tumor-killing activity of this produced nanoparticle (Fang et al., 2020).

Also, lipid-polymer hybrid nanoparticles (LPNs) were designed to increase the therapeutic efficiency of nanoparticles, which have been used to deliver DTX and curcumin drugs (Chen et al., 2020). Considering the role of DTX in preventing and suppressing the growth of tumor cells and the role of curcumin in modulating the immune system’s responses (Abbaspour-Aghdam et al., 2022), it seems that these nanoparticles can help treat PC. In this study, different combinations of polymer nanoparticles and drugs have been used; the highest efficiency is related to the group carrying both drugs DTX and curcumin, which shows the synergistic effect of these two drugs in preventing the growth of PC3 tumor cells *in vitro* (Yan et al., 2016). *In vivo* studies also confirm the results of *in vitro* studies and show that these PNPs can prevent tumor growth induced by injecting PC3 cells in mice without systemic toxicity (Yan et al., 2016). In the continuation of this study, this research group has used a peptide called EGFR peptide (GE11) for targeted delivery of nanoparticles containing DTX and curcumin, which are pH-sensitive for treating PC. The *in vitro* and *in vivo* results show that GE11 leads to the targeted delivery of drugs encapsulated in nanoparticles and, through synergism, is a promising system for treating PC (Yan et al., 2017).

In addition to chemotherapy drugs and herbal drugs that can be transferred to PCCs by PNPs and change their characteristics, in some studies, PNPs have been used to transfer microRNAs (Lee et al., 2019). miRNAs are essential in regulating cell responses, metabolism, cellular stress, inflammatory responses, etc. (Ambros, 2004; Krützfeldt et al., 2006; Sun and Lai, 2013). In the study of Conte et al. (2020), PHB-PEI NPs loaded with miR-124 were used for the treatment of PC *in vitro* and *in vivo*. miR-124 modulates the expression of carnitine palmitoyl transferase 1A (CPT1A) at the post-transcriptional level and impairs the ability of androgen-independent prostate cancer (PC3) cells to metabolize lipid substrates fully (Conte et al., 2020). The results of this study show that PNPs synthesized by increasing the expression of miR-124 reduce the signs of tumorigenesis, such as motility, cell proliferation, and colony formation in PC3 cells and the mouse tumor model induced by these cells (Conte et al., 2020).

### 3.2 Inorganic material-based system

According to the desired source for producing NPs, there are two production methods. These methods generally include the mechanism from top to bottom and the method from bottom to top (Jamkhande et al., 2019). Among the top-down production methods of metallic nanoparticles, we can mention methods based on mechanical milling, laser ablation, and sputtering (Nadagouda et al., 2011). Also, bottom-up methods include liquid, solid, gas, and biological methods (Mukherjee et al., 2001; Nadagouda et al., 2011). Some metals have inherent properties, including antimicrobial properties, whose production in nano size leads to an increase in their therapeutic potential. Gold and silver are among the metals used in biomedicine, and they are widely used (Alaqad and Saleh, 2016; Slepička et al., 2019). The results of many reports have shown that, for example, gold nanoparticles have immunomodulatory and antitumor properties (Joseph et al., 2013; Elbagory et al., 2019). Among MNPs, gold-based nanoparticles (AuNPs) are more beneficial due to their properties.

The therapeutic potential of AuNPs has been revealed in various studies. In a study conducted in 2020 by Mangadlao et al. (2018), the specific membrane antigen expressed by prostate cancer cells (PSMA-1) was attached to AuNPs along with a fluorescent photodynamic therapy (PDT) drug for the treatment of PC used *in vivo* and *in vitro*. The results of the experimental phase of this study show that these nanoparticles have accumulated in PC3 cells. After PC3 cells are exposed to light at different doses, tumor cells are killed, which indicates active targeting followed by delivery of NPs. Also, the *in vivo* results show the improvement of PSMA-expressing tumors in PC model mice 14 days after injecting this nanoparticle (Mangadlao et al., 2018). In addition to tumor-specific antigens that can be attached to AuNPs, some tumor-associated antigens can also be placed on their surface and help treat PC (Westdorp et al., 2014). A study conducted by Shukla et al. (2012) showed that therapeutic gold nanoparticles derived from the Au-198 isotope and prostate tumor-specific epigallocatechin-gallate (EGCg) lead to the treatment of prostate cancer. EGCg can bind to Laminin67R, which is highly expressed by tumor cells and leads to the activation of gold nanoparticles (Shukla et al., 2012). The study results show that 72% of these injected NPs are preserved for 24 h in the body of mice with PC, which leads to a decrease in the volume of tumors by 80% (Shukla et al., 2012).

In addition to the direct effects of gold nanoparticles on cancer cells, this type of treatment can increase the sensitivity of tumor cells to radiotherapy (Zhang et al., 2008; Roa et al., 2009). Due to the radiosensitizing feature of AuNPs, they are used to improve the efficiency of X-ray radiation therapy (Hainfeld et al., 2006). Also, for the effects of X-rays to be more on the points related to the presence of tumor cells, the accumulation of nanoparticles that have reached the tumor site in a targeted manner leads to an increase in the effects of radiotherapy on tumor cells. For example, in the study conducted by Luo et al. (2019) AuNPs coated with PSMA, a prostate tumor-specific antigen, lead to the specific accumulation of AuNPs in the tumor site and increase the efficiency of radiotherapy (Hara et al., 2021).

Also, given that AuNPs strongly scatter light at or near the surface plasmon resonance (SPR) wavelength region, AuNPs combined with dynamic light scattering (DLS) detection, an easy NPs immunoassay to detect and analyze a serum protein biomarker has been developed for PC patients (Khoo et al., 2017). In addition to X-rays, it has been shown that using AuNPs *in vivo* can increase the efficiency of megavoltage radiation. In this study, AuNPs were coated with goserelin, an analog of LHRH that binds to LHRHR (Wolfe et al., 2015). Since PCCs highly express LHRHR, this leads to the targeted migration of AuNPs in the body towards PCs. In another study, AuNPs loaded with soybean genistein (Gen) were used to examine the proliferation and characteristics of PC3 *in vitro* (Wolfe et al., 2015).

Due to the inherent properties of carbon, CNPs have electron affinity, electrical conductivity, versatility, high strength, and electrical conductivity (Srivastava et al., 2017; Lisik and Krokosz, 2021). Generally, CNPs are divided into two categories: carbon nanotubes (CNTs) and fullerenes such as (for example, nanospheres) (Astefanei et al., 2015). Based on the number of carbons in the structure, CNPs can be divided into different classes. As it is clear from the name of CNTs, they have a long tubular structure with a diameter of 1–2 nm (Aqel et al., 2012; Ibrahim, 2013). In addition, various studies have shown that these structures are biocompatible and have a high ability to penetrate tumor cells (Smart et al., 2006). These structures can also be used to diagnose and treat PC (Murugesan and Raman, 2022). While CNTs are mainly used in cancer treatment, few studies have focused on diagnosing and treating PC. In addition to the fact that CNPs can be used for drug delivery, they can increase the therapeutic efficiency of some drugs, including chemotherapy drugs.

In the study conducted by Erdmann et al. (2017) CNTs have been combined with chemotherapy drugs such as docetaxel and mitomycin C (MMC) to investigate their effect *in vitro*. Proliferation, survival, and apoptosis rates of DU-145 PCa cells, a small cell related to prostate cancer, were investigated. The results show that the combined use of CNTs, DTX, and MMC increases their potential compared to their single-use (Erdmann et al., 2017). It has also been shown that CNTs increase drug delivery to cancer cells due to their biocompatibility and integration with cells. In another study, Fe3O4@C nanoparticles contain a carbon cortex and transport ascorbic acid (AA) by binding to its cortex (Fe3O4@C-AA) (An et al., 2013). The semi-graphitic carbon Fe3O4@C facilitates the transfer of electrons and decomposition of H2O2 and the production of reactive and toxic free hydroxyl radicals for cancer cells (An et al., 2013). The results of this study show the synergistic effects of NPs and AA and decrease the viability of PC3 cells. In this study, HEK293 cells were used to investigate the safety of these produced NPs, and it was shown that due to the high ability of normal cells to deal with ROS, the cytotoxic effects of these NPs are low in these cells. Therefore, it can be said that this produced NP can specifically affect tumor cells (An et al., 2013). Examining the effect mechanism of NPs on prostate cancer cells shows that they do this through a multifactorial mechanism. On the one hand, they can affect the phosphorylation of the Akt enzyme, and on the other hand, they affect mast cells by affecting the pathways related to translation, including 4E-BP1. Western blot analysis of 4E-BP1 *in vitro* and *in vivo* shows the reduction of this factor in PC3 cells and PCa tissue samples from nude BALB/c mice (Dong et al., 2020).

In addition to the role of CNTs in treating PC, it seems that they can be used to increase the targeted ultrasound contrast agent (Delogu et al., 2012; Cai et al., 2013). Considering the vital role of early detection of PC in the life of patients and deciding on different treatments, it is essential to expand the methods that help it (Carter et al., 2013; Zhou et al., 2017). Current methods based on visual examination as well as ultrasound imaging have the possibility of increasing efficiency (Cookson, 2001; Thompson and Ankerst, 2007). In a study published by Gu et al. (2018), PEG-coated CNTs attached to PSMA were used to improve the effectiveness of PC detection. *In vitro* studies show that these NPs are easily absorbed by tumor cells and have high biocompatibility. The results of this study show that in BALB/c nude mice, the PC model receiving these NPs shows better US imaging visual contrast than traditional methods (Gu et al., 2018).

In some other studies, CNPs have been used simultaneously for diagnosis and treatment. In these studies, very complex NPs based on CNTs have been used. To quickly detect the biodistribution of these NPs, they are attached to fluorescein isothiocyanate (FITC). Polyethylenimine (PEI) acts as a bridge for the covalent attachment of FITC to CNTs in this complex structure. Also, a monoclonal antibody against prostate cancer stem cell-specific antigen (PSCA) is attached to this structure to be specifically attached to PCCs (Wu et al., 2014). Investigations of this produced NP show its biocompatibility in the body and the laboratory. The use of confocal luminescence imaging, ultrasound imaging, and combined flow cytometry in the *in vitro* and *in vivo* conditions shows the specific attachment of these NPs to prostate cancer cells, which can be used in diagnosing PC and a targeted contrast agent (Wu et al., 2014). In addition, this complex complex can be used as a carrier of various drugs to suppress tumor growth and help cell survival and cancer models in animals.

### 3.3 Bioinspired system

Exosomes are nano-sized vesicles produced by different cells and perform other functions in cell-to-cell communication (Yong et al., 2019). These vesicles have different therapeutic potentials, including the potential to deliver different drugs (Hazrati et al., 2023). Various drugs can be loaded into exosomes and transferred to target cells through methods such as sonication, electroporation, and incubation at room temperature (Tanziela et al., 2020). In addition, exosomes produced from tumor cells can play a role in pathogenesis and progression (Wolfers et al., 2001). Exosomes derived from cancer cells, by transferring various cargoes to the cells in the TME, lead to increased proliferation of cancer cells, suppression of immune system responses, increased angiogenesis, and tumor progression (Yu et al., 2015; Whiteside, 2016). These exosomes can help EMT and increase metastasis in cancer cells by transferring the integrin α2 subunit and increasing the ERK effector (Gaballa et al., 2020). In addition, it has been shown that exosomes produced from PCCs can help the chemoresistance of tumor cells by introducing chemotherapeutic drugs into the exosome and sending it to the pore from the cell (Table 3) (Mashouri et al., 2019; Milman et al., 2019).

**TABLE 3:** Exosomes as a tumor-promoting vehicle in prostate cancer and their potential for use as diagnostic markers.

Exosome cargo type	Donor cells or tissue	Affected cell	Tumor promoting mechanism	Ref
Prostate-specific G-protein-coupled receptor	PC3	hFOB1.19	↑ EMT by promoting migration, invasion, stemness	Li et al. (2020a)
Integrin alpha 2 subunit	PC3	LNCaP	↑ EMT	Gaballa et al. (2020)
miRNA-26a	LNCaP, PC-3	LNCaP, PC-3	↑ EMT	Wang et al. (2019)
miR-217,miR-23b-3p	PC-3, DU145	PC-3, DU145	↑ EMT and tumor cell proliferation	Zhou et al. (2020)
CIRC_0081234	MDA-PCA-2b	22RV1, DU145	↑ EMT by promoting migration, invasion, stemness	Zhang et al. (2022)
FAK, c-Src, GRK, IGF-IR	PC-3, DU145, and C4-2B	PC-3, DU145, and C4-2B	↑ Tumor growth and angiogenesis	DeRita et al. (2017)
Phosphoglycerate mutase 1	C4-2, DU145, and PC-3	HUVECs, RWPE-1	1. ↑ Podosome formation and neovascular sprouting in HUVECs 2. ↑ Lung metastasis in nude mice	Luo et al. (2023)
miR-27a-3p	PC3	Human umbilical vein endothelial cells	↑ Angiogenesis in endothelial cells	Prigol et al. (2021)
miR-142–3p, miR-142–5p and miR-223–3p	Semen	NA	↑ PC diagnosis/prognosis efficiency	Barceló et al. (2019)
Exosome	PCCs	NK cell and T CD8^+^	1. ↓ NKG2D expression on circulating NK and CD8^+^ T cells 2. ↑ Immune suppression and tumor escape	Lundholm et al. (2014)
circ_0044516	Prostate cancer tissue	NA	1. Downregulate miR-29a-3p expression 2. ↑ PCC survival and metastasis	Li et al. (2020b)
miR-141–3p	MDA-PCA-2b	MDA-PCA-2b	1. ↑ Osteoprotegerin OPG expression 2. ↑ Bone metastasis	Ye et al. (2017)

Regarding PC, exosomes derived from cancer cells can be seen in blood and urine, which is a sign of metastasis (Tavoosidana et al., 2011; Øverbye et al., 2015). Considering that exosomes derived from tumor cells carry tumor-specific antigens, the isolation, and examination of the presence of these antigens in exosomes derived from the blood and urine of patients (for example, PSA) can help in the diagnosis of prostate cancer (Liu et al., 2014; Logozzi et al., 2017). Exosome miRNAs are also one of the leading indicators of cancer diagnosis by exosomes. Among the exosomal miRNAs used to diagnose PC, miR-375 and miR-1290 can be mentioned (Huang et al., 2015). Hempnin exosomes derived from PCCs have a large amount of αvβ3 on their surface (Krishn et al., 2019). Also, the contents of exosomes derived from PCCs can help predict treatment outcomes with different drugs. For example, the presence of B7-H3 (CD276) and HSP72 indicates the treatment of this cancer with radiotherapy (Hurwitz et al., 2010; Erozenci et al., 2019).

As mentioned, in addition to diagnosis, exosomes help treat PC (Pan et al., 2017). However, various studies show that using intact exosomes has low efficiency in cytotoxicity against tumor cells (Batrakova and Kim, 2015). They are used in different treatment platforms, and drug delivery is one of the most essential (Wang et al., 2021). Exosomes loaded with paclitaxel were used in a study by Saari et al. These exosomes were incubated *in vitro* with LNCaP and PC-3 cells, and the results show that they have cytotoxic effects on these cells. Some other studies have focused on targeting exosomes (Saari et al., 2015). In another study, Exo-PMA/Fe-HSA@DOX nanocarriers were used to treat prostate cancer (Pan et al., 2021). These exosomes are isolated from the urine of patients and bind specifically to PCCs. Also, these exosomes are loaded with doxorubicin, a chemotherapy drug, to increase its therapeutic potential (Pan et al., 2021). The results of this study show that these exosomes have successfully penetrated the tumor cells by affecting the signaling of the epidermal growth factor receptor (EGFR) and inhibiting its downstream pathway, i.e., AKT/NF-kB/IkB from the prevent tumor cell growth (Pan et al., 2021). In other studies, mesenchymal stem cell-derived exosomes encapsulated by iron nanoparticles were used to incubate with PC3 cells (Altanerova et al., 2017). According to the characteristics of exosomes derived from mesenchymal stem cells (Chenari et al., 2023) and using heat therapy caused by an external alternating magnetic field, the toxicity of this exosome and its cytotoxic effects on PC3 cells were proven (Altanerova et al., 2017). In addition to the above, to target the migration and binding of exosomes, PSMA targeting protein was attached to the membrane of exosomes so that they could specifically bind to PSMA^+^ prostate cancer cells, including C4-2B and LNCaP cells (Severic et al., 2021). Due to their intrinsic potential in killing tumor cells, exosomes derived from immune system cells have retained their cell characteristics and can affect cancer cells (Hazrati et al., 2022). Exosomes derived from dendritic cells isolated from patients are a rich source of tumor antigens that can activate the responses of TCD8^+^ cells (André et al., 2004). It has also been shown that exosomes derived from M1 macrophages have the inherent potential to kill and phagocytose tumor cells (Cui et al., 2022).

### 3.4 Lipid-based system

Because NPs of different species have a different nature compared to cells, in some cases, their delivery to the cell becomes difficult, so replacing nanomedicines based on the characteristics of the target cell is very important. Different vesicles as drug carriers in PC treatment are used, among which exosomes, liposomes, and micelles can be mentioned. Also, new studies have used the term nanobubbles as a drug carrier. Nanovesicles (NVs) create a safe platform for drug transfer to the target cell so that enzymes and environmental conditions do not affect the characteristics of the drug before reaching the target cell. In addition, because, in most cases, the membrane of these vesicles is very similar to the biological membranes of cells, they merge with the target cells with high efficiency and transfer the desired drug. In addition, because cancer cells produce exosomes in abundance, they can be used as diagnostic factors for prostate cancer.

#### 3.4.1 Liposomes

**FIGURE 2:**
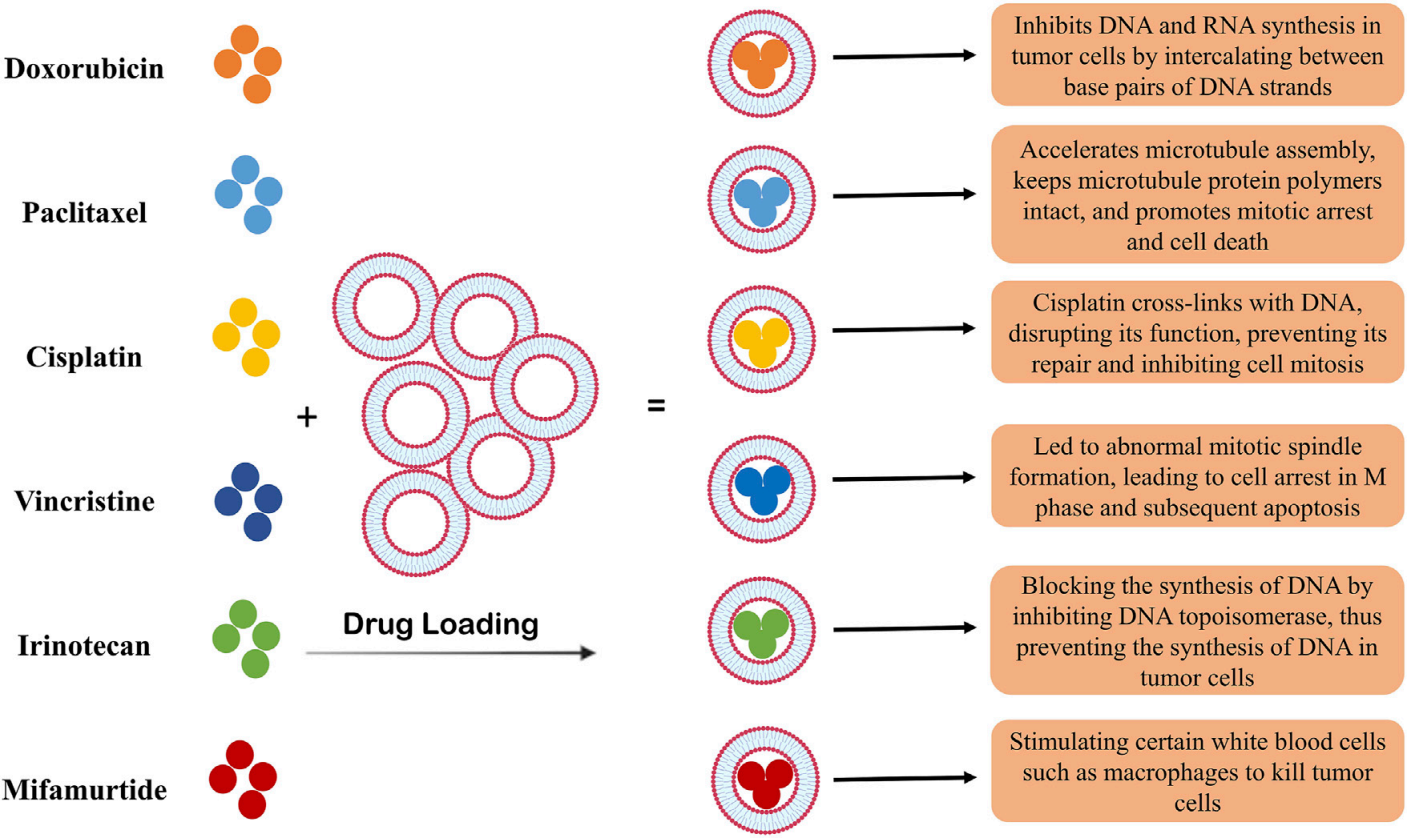
The role of liposomes as carriers of chemotherapy drugs. Liposomes have a high capacity to encapsulate different drugs and have been used as a carrier in cancer treatment for many years. In the treatment of prostate cancer, as shown in the figure, different types of chemotherapy drugs have been used. The use of liposomes led to the slow release of the drug and the increase in the efficacy of the drugs, which is associated with a decrease in the survival of tumor cells and an increase in their apoptosis.

Liposomes are vesicles consisting of synthetic lipid bilayers widely used in nanomedicine (Beltrán-Gracia et al., 2019; Rommasi and Esfandiari, 2021). In the therapeutic applications of liposomes to deliver drugs to tumor cells, they are usually targeted, and the molecules that lead to the specific identification of tumor cells are expressed on their surface (Caracciolo, 2018; El-Readi and Althubiti, 2019). Clinical studies related to liposome application in the treatment of prostate cancer have been conducted with only doxorubicin in them (Sawpari et al., 2023). It has been shown that the pegylation of liposomes leads to their stability in the body and blood flow for a long time and increases the effects of doxorubicin (Figure 2) (Solomon and Gabizon, 2008; Kroon et al., 2014).

On the other hand, liposomes encapsulating other drugs are in the laboratory stage or animal studies. For example, liposomes encapsulated with curcumin and resveratrol have significantly suppressed tumor growth in B6C3F1/J PC model mice (Narayanan et al., 2009). Also, animal studies using cationic liposomes encapsulated with PTX show their high potential in suppressing the growth of prostate-related tumors (Narayanan et al., 2009).

In a study, to increase the efficiency and targeted binding of liposomes to cancer cells, a three-amino acid-containing peptide that included arginine-glycine-aspartic acid-tyrosine-lysine cyclic peptide (cRGDyk) was placed on the surface of liposomes (Wang et al., 2014). This peptide ligand is an integrin to αv and β3 components, which plays a vital role in bone metastasis of cancer cells (Nemeth et al., 2003; McCabe et al., 2007). Also, these exosomes are encapsulated with cisplatin. Cisplatin is a chemotherapy drug that binds to double-stranded DNA and inhibits DNA synthesis and cancer cell proliferation. The results of the study by Wang et al. show that cisplatin loaded from cRGDyk liposomes with high potential is absorbed by prostate cancer cells *in vitro* (Wang et al., 2014). Although the results of the experimental phase have been promising, the results of the *in vivo* phase of using single cisplatin show that this drug does not affect the survival of mice with prostate cancer (Gumulec et al., 2014). However, the results of cisplatin loaded in targeted liposomes show their accumulation in bone, prevention of metastasis induced by PC, the synergistic antitumor activity of the drug and the ligand present on the surface of liposomes and αvβ3, reduction metastasis in affected mice, and increase their overall survival (Wang et al., 2014). Therefore, considering that the inhibition of metastasis is significant in treating invasive tumors, it seems that using such systems could have a promising future in the clinic.

In another study, paclitaxel liposome-loaded was used to target neovascularization in PC (Lee et al., 2012). As mentioned before, due to the presence of a positive charge in cationic liposomes and the presence of a negative charge in the plasma membrane of immature endothelial cells, these vesicles are easily absorbed by endothelial cells and can release the loaded drug into the cell (Qi et al., 2016; Rayamajhi et al., 2020). The results of this study show that PTX-encapsulated liposomes, compared to PTX alone, have reduced the size and number of tumors and prevented metastasis and the production of new blood vessels in model mice (Bode et al., 2009).

**FIGURE 3:**
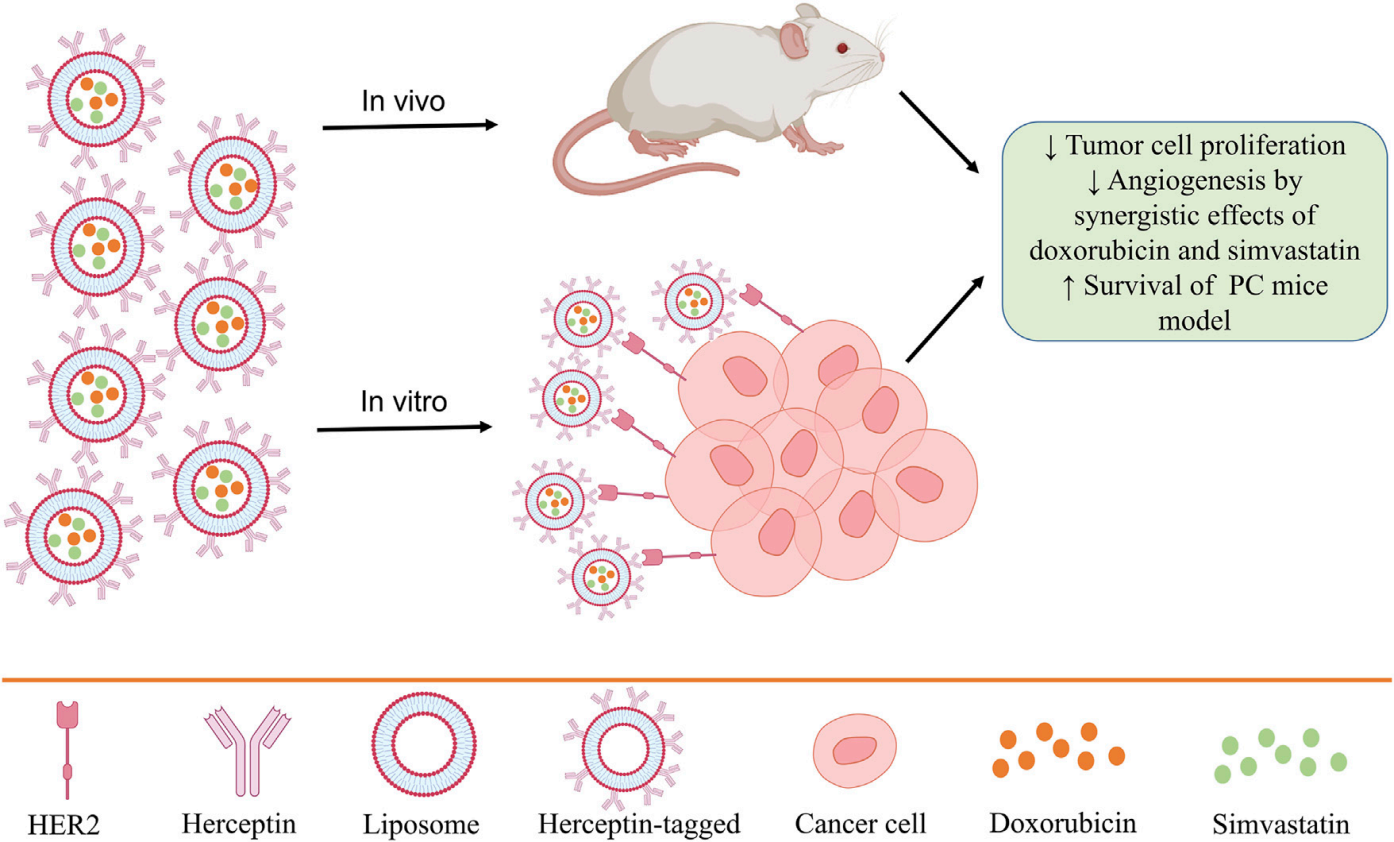
Therapeutic use of Herceptin-tagged liposomes loaded with doxorubicin and simvastatin.

Knowing the information and characteristics of tumor cells also helps in their treatment. Because PCCs express epidermal growth factor receptors, including HER2, using a system that can identify them can help treat prostate tumors (Edwards et al., 2004). In some studies, the efficacy of Herceptin (trastuzumab), which is an antibody against HER2, leads to the regression of prostate and breast tumors (Agus et al., 1999; Ziada et al., 2004). In this regard, a study used Herceptin-tagged engineered liposomes for PC treatment. Also, due to the high potential of liposomes in drug delivery, two drugs, doxorubicin, and simvastatin, were simultaneously loaded in these liposomes, and they have been used in both *in vitro* and *in vivo* diseases to investigate therapeutic performance (Li et al., 2019). The results show that due to the high expression of HER2 on the surface of PC3 cancer cells, the use of these liposomes *in vitro* and *in vivo* leads to the reduction of tumor cell proliferation, the synergistic effects of doxorubicin and simvastatin in preventing angiogenesis and increasing the survival of mice (Figure 3) (Li et al., 2019).

#### 3.4.2 Micelles

Micelles comprise a phospholipid layer membrane, usually connected to different materials, including polymers (Gong et al., 2012). Genexol-Polymeric micelles (PolM) were first approved in 2007 by the US Food and Drug Administration (FDA) as carriers of chemotherapy drugs (Genexol) (Oerlemans et al., 2010; Miyata et al., 2011). Their favorable characteristics, including the presence of lipophilic groups, the possibility of recognizing them by different receptors expressed by cells, and the possibility of integrating them with physiological membranes, have made them a valuable tool in drug delivery (Gong et al., 2012). Micelles’ structure can be divided into two parts. The aqueous part, the outer part, is the main factor in their pharmacokinetic behavior, and the inner part, or the core, is hydrophilic and is the place of drug loading. In different studies, different formulations have been defined for the production of mucilages (Ahmad et al., 2014). PolM is usually used to transfer chemotherapy drugs and increase their therapeutic efficiency (Al-Achi and Lawrence, 2013).

As mentioned, cabazitaxel can help treat various cancers by inhibiting tubulins (Galsky et al., 2010). However, due to their low solubility and the possibility of side effects, these drugs should be used in targeted delivery systems to incresment their therapeutic applications (Michielsen et al., 2011). In the study by Ashutosh Barve and their colleagues, they designed a PolM, whose copolymer has a degradable peptide by the enzyme. The peptide used in this drug delivery system is degraded by matrix metalloproteinase 2 (MMP-2) (Lévesque and Shoichet, 2007). Considering that MMP-2 is abundantly produced in prostate tumor tissue (Trudel et al., 2003), it seems that the use of this system can lead to the targeted delivery of the drug loaded in these micelles, i.e., cabazitaxel. An important point about this type of micelle produced is that the release of cabazitaxel from the micelle depends on the cleavage of the MMP-2 responsive peptide. Also, a different kind of PolM designed by this group that contains a ligand associated with prostate cancer, namely PSMA, was used in them (Barve et al., 2020). The binding of the receptor-ligand leads to the release of the drug to the cancer cells. The results of this study show that the enzyme-based micellar system can significantly increase the drug delivery efficiency compared to the ligand-based micellar, intact micellar, and cabazitaxel in 3-D tumor spheroids (Barve et al., 2020). Also, the cytotoxic activity of chemotherapy drugs on tumor cells in micelles based on enzyme regulation was significantly higher than in other groups (Barve et al., 2020). In another study, PolM conjugated with tumor-specific aptamer was used. This micelle has different layers, from the inside to the outside, including 1) an H40 core, 2) a drug loaded in the micelle, which is doxorubicin, 3) a polymer including PLA + PEG, 4) aptamer that binds to PSMA. The results of this study show that these types of micelles can improve the biological distribution of the drug in both *in vitro* and *in vivo* conditions and also deliver their contents, DOX, to cancer cells with higher efficiency (Xu et al., 2013). Molecular investigations in this study show increased cleavage and conversion of inactive form to active form in PARP and Caspase 3 molecules. Also, the expression level of Bax, which is a pro-apoptotic protein, increases in PCCs. The level of Bcl-2 as an anti-apoptotic protein and P21 and P27, which lead to cell cycle arrest, increases after treatment (Xu et al., 2013). Therefore, using this drug delivery system generally leads to an increase in apoptosis and cell cycle arrest in cancer cells.

In other studies, two drugs loaded micelles were used to treat prostate cancer. In the study conducted by Feng Lin in 2020, DTX and rubon (RUB) were utilized in pH and glutathione (GSH) micelles (Lin et al., 2019). Regarding the use of two drugs, the combined mechanisms that they regulate are both involved in inhibiting tumor production (Lin et al., 2019). The results of this study show that this designed drug system can increase the expression of miR-34a by prostate cancer stem cells and prevent their growth (Lin et al., 2019). They developed another PEO-PCL-based system that carries anti-nucleostemin and docetaxel siRNA and binds to the DCL ligand. The *in vitro* results show that this drug delivery system leads to an increase in the apoptosis of PSMA^+^ tumor cells, a significant decrease in nucleostemin expression, and an arrest in the G1/S and G2/M mitotic cycle of tumor cells.

Therefore, different drug delivery combinations using micelles were used, including two chemotherapy drugs and chemotherapy drugs and gene therapy mediators. Various mechanisms can also be used to target drug delivery by micelles, which can be used in systems based on enzymatic decomposition, specific ligands, and aptamers.

#### 3.4.3 Nanobubbles

Nanobubbles are gas-filled structures used for ultrasound agents (US) to increase the contrast of prostate cancer MRI images (Song et al., 2020). Usually, this structure plays a role in treating and diagnosing tumors (Jin et al., 2022). Nanobubbles, like micelles, are made of a phospholipid layer and several other layers that help maintain their structure and targeted identification (Foudas et al., 2023). Inside these vesicle-like structures were gases such as octafluoropropane (C3F8) (Perera et al., 2019). They perform different actions depending on the type of gas inside the nanobubbles. For example, considering hypoxia’s vital role in TME, using nanobubbles containing oxygen can help treat tumors by eliminating hypoxia (Song et al., 2020). Also, attaching specific receptors makes it possible to target nanobubbles to the intended tissue. Regarding PC, in various studies, by attaching A10-3.2 aptamers to nanobubbles (targeting PSMA), they are targeted to migrate to the prostate tumor site (Fan et al., 2016).

In the study by Wu et al. (2017) polymeric nanobubbles containing PLGA were used, which bind to PSMA expressed by prostate cancer cells by having A10-3.2 aptamers. Also, PTX was loaded in these nanobubbles through water/oil/water double emulsion. Fluorescent microscope studies confirm the synthesis and attachment of these nanobubbles to the target cells. The results of this study show that these structures slowly deliver the drug in high concentration to the tumor cells *in vivo*. In addition, these nanobubbles can lead to the apoptosis of tumor cells by 50% more than the single use of PTX *in vitro* (Wu et al., 2017). Also, their use *in vivo* leads to increased survival and overall survival of mice in the prostate tumor xenograft model. It was also shown in this study that the use of these nanobubbles can lead to an increase in the quality and contrast of photos related to fluorescent microscopy and ultrasound imaging. Therefore, this technology can use high-quality images to check the treatment process or disease. Also, by combining nanobubbles with chemotherapy drugs, their potential can be used for targeted prostate tumor treatment (Wu et al., 2017). In another study, nanobubbles containing A10-3.2 aptamers were used. The difference between this study and the previous one is that siRNA loaded in nanobubbles was used instead of usual chemotherapy drugs (Wu et al., 2018). This siRNA is against the Forkhead box M1 (FoxM1) transcription factor, which plays a vital role in the development and proliferation of tumor cells. Fluorescence and flow cytometry studies show that this nanobubble is attached to the PSMA of positive LNCaP cells and has transferred the loaded siRNA to them. siFoxM1-Apt-CNBs combined with ultrasound-mediated nanobubble destruction (UMND) significantly improved transfection efficiency, cell apoptosis, and cell cycle arrest *in vitro* while downregulating FoxM1 expression (Wu et al., 2018).

Another category of nanobubbles, known as magnetic nanobubbles, is also used for molecular imaging of prostate cancer (Zhu et al., 2020). Like therapeutic nanobubbles, magnetic nanobubbles have been connected to PSMA ligands to be specifically linked to prostate tumor cells. The results of these studies show that they can increase the effectiveness and specificity of MRI/US images (Zhu et al., 2020).

Like other vesicle-based nanomedicines, nanobubbles can also act multifunctionally and help treat prostate cancer by loading two or more different drugs. In a study by Lan et al. (2020), nanobubbles containing indocyanine green and paclitaxel were used for prostate cancer imaging and treatment. Like other studies, the results show the targeted connection and therapeutic performance with high efficiency of these nanobubbles.

## 4 Conclusion and future perspective

Due to the positive features of treatments based on nanomaterials, a new branch called nanomedicine was born to treat and diagnose tumors. Nanomedicine in treating tumors has many different and wide branches; in this study, we discuss nanoparticles and nanovesicles, their various types, and their therapeutic potential. Considering that chemotherapy drugs have toxicity and side effects on other organs, are not targeted, and cannot be detected, we need a drug delivery platform to use them. NPs and nanovesicles, in addition to their direct therapeutic effects in the treatment of PC, have a high potential for the targeted delivery of chemotherapy drugs. Different studies have used new technologies such as aptamers, specific tumor cell ligands, and antibodies attached to NPs and NVs to target them for the treatment of prostate cancer (Table 4). In addition to their role in treatment, they can be used in tumor diagnosis. In addition to exosomes being identified as NVs produced from tumor cells in blood and body fluids, other nano-based materials, including NPs and especially nanobubbles, can be used in tumor diagnosis. Nanobubbles can improve the contrast of MRI images and help to better diagnose the tumor tissue and its size in different stages and after treatment. The results of various studies presented in this article have been encouraging and are expected to revolutionize tumor treatment. However, it should be noted that most of these studies are in the laboratory phase and animal studies, and few of them have made their way to the clinic. Among the reasons and limitations that lead to the lack of translation of studies on the use of nanomedicine in the treatment of PC are the difficulty of homogenous synthesis of NPs, the impossibility of controlling their biodistribution, their small size that allows them to pass through the blood-brain barrier, and side effects. He pointed out the possibility of their accumulation in places with tiny capillaries, including joints and kidneys, and the lack of decomposition of some of them in the body. Also, in the case of exosomes, it has been shown that in their intact state, they have low efficacy for therapeutic applications and must undergo engineering, which makes their preparation process time-consuming and expensive. In addition, a good manufacturing process (GMP) must be observed to obtain exosomes with therapeutic applications, which increases the difficulty of preparing exosome-based treatments for prostate cancer in clinical trials. It seems that the combined use of immunotherapies (such as CAR-T cell, cytokines and vaccines) and nanomedicines based on NVs and NPs can help treat patients. Also, considering the importance of microbiota in maintaining the homeostasis of the immune system and the function of various organs, it seems that using microbiota-improving methods in combination with nanomedicine-based treatments can increase their effectiveness.

**TABLE 4:** Example of NPs application in clinical trials.

Study name	Study type	Estimated enrollment	Combined therapy	Phase	Therapeutic or diagnostic	NTC number
Magnetic nanoparticle thermoablation-retention and maintenance in the prostate	Interventional	12	Not applicable	Early phase 1	Therapeutic	NCT02033447
An extension study MRI/US fusion imaging and biopsy in combination with nanoparticle directed focal therapy for ablation of prostate tissue	Interventional	60	Not applicable	Not applicable	Diagnostic	NCT04240639
Nanoparticles and EBRT or EBRT with brachytherapy in the treatment of prostate adenocarcinoma	Interventional	5	Radiotherapy	Phase 1 Phase 2	Therapeutic	NCT02805894
MRI/US fusion imaging and biopsy in combination with nanoparticle directed focal therapy for ablation of prostate tissue	Interventional	45	Laser irradiation	Not applicable	Diagnostic	NCT02680535
Combining CRLX101, a nanoparticle camptothecin, with enzalutamide in people with progressive metastatic castration resistant prostate cancer following prior enzalutamide treatment	Interventional	4	Enzalutamide	Phase 2	Therapeutic	NCT03531827
The use of nanoparticles to guide the surgical treatment of prostate cancer	Interventional	10	Not applicable	Phase 1	Diagnostic	NCT04167969
